# La evaluación de tecnologías sanitarias en el Ministerio de Salud Pública de Ecuador como herramienta para la compra de medicamentos entre 2012 y 2015

**DOI:** 10.26633/RPSP.2017.50

**Published:** 2017-05-15

**Authors:** Luciana Armijos, Santiago Escalante, Tatiana Villacrés

**Affiliations:** 1 Ministerio de Salud Pública La correspondencia se debe dirigir a LucianaArmijos QuitoPichincha Ecuador Ministerio de Salud Pública, Quito, Pichincha, Ecuador. La correspondencia se debe dirigir a LucianaArmijos.; 2 Pontificia Universidad Católica del Ecuador Pontificia Universidad Católica del Ecuador Quito Ecuador Pontificia Universidad Católica del Ecuador, Quito, Ecuador.

**Keywords:** Evaluación de la tecnología biomédica, tecnologías médicas, políticas públicas de salud, toma de decisiones, medicamentos, Ecuador, Technology assessment, biomedical, public health policy, decision making, pharmaceutical preparations, Ecuador

## Abstract

**Objetivo.:**

Conocer el uso de la evaluación de tecnologías sanitarias (ETS) en la toma de decisiones del Ministerio de Salud Pública (MSP) del Ecuador para la compra de medicamentos que no se encuentran en el Cuadro Nacional de Medicamentos Básicos (CNMB).

**Métodos.:**

Con la información de las bases de datos de la Dirección de Inteligencia de la Salud (DIS) y la Dirección Nacional de Medicamentos y Dispositivos Médicos (DNMDM), se compararon las decisiones tomadas por ambas instancias, para conocer el uso y la congruencia de los informes de ETS en las decisiones de compra de los medicamentos no incluidos en el CNMB.

**Resultados.:**

Entre 2012 y 2015, se han elaborado 227 informes, de los cuales 87 corresponden a medicamentos, 36 a dispositivos, 29 a procedimientos médicos, 34 a programas sanitarios, y 41 a otras tecnologías médicas. De los informes de medicamentos, 59 fueron solicitados por la DNM. La concordancia entre las decisiones tomadas por las dos direcciones que participan en el proceso alcanzó 80%.

**Conclusiones.:**

La ETS se inició en el MSP en 2012 a través de la DIS. Considerando que la mayoría de informes evalúan medicamentos, es indispensable que se desarrollen informes para otros tipos de tecnologías médicas y que se difunda al máximo su desarrollo y uso. A pesar de que el nivel de concordancia entre las decisiones es elevado, es importante seguir mejorando el alcance y la calidad de los informes, así como monitorizar la incorporación y difusión de las tecnologías autorizadas y financiadas para conocer la efectividad y el impacto de la ETS en Ecuador.

## Abstract (en)

***Objective*.:**

Learn how the Ministry of Public Health (MSP, the Spanish acronym) of Ecuador uses health technology assessment (HTA) in decision-making on the purchase of drugs that are not on the National List of Essential Medicines (NLEM).

***Methods*.:**

Information from databases of the Health Intelligence Directorate (DIS, the Spanish acronym) and the National Directorate of Drugs and Medical Devices (DNMDM, the Spanish acronym), was used to compare decisions made by both entities, to learn about the use and consistency of HTA reports in decisions on purchasing drugs not included in the NLEM.

***Results*.:**

From 2012 to 2015, 227 reports were issued, of which 87 cover drugs; 36, devices; 29: medical procedures; 34: health programs; and 41: other medical technologies. The DNMDM requested 59 of the reports on drugs. There was 80% agreement in decisions made by the two directorates that participate in the process.

***Conclusions*.:**

The MSP, through the DIS, began using HTA in 2012. Given that the majority of reports evaluate drugs, it is essential that reports be prepared for other types of medical technologies and that they be prepared and used as widely as possible. Despite a high level of agreement in decisions, it is important to continue to improve the reports’ scope and quality, and to monitor adoption and dissemination of authorized and funded technologies to learn the effectiveness and impact of HTA in Ecuador.

Desde inicios de la década de los noventa, la mayor parte de los países de América Latina han entrado en un proceso de reforma de sus sistemas y servicios de salud. Su propósito principal consiste en aumentar la equidad en sus prestaciones, así como la eficiencia de su gestión y sus actuaciones para satisfacer las necesidades de salud de la población. (1, 2). La reforma del sector salud en Ecuador trae consigo cambios y desafíos al considerar la salud como un derecho constitucional garantizado por el Estado. En este contexto, surge la necesidad de utilizar herramientas que permitan tomar decisiones sustentadas en pruebas científicas de calidad, para asegurar la sostenibilidad de los procesos y la financiera del sistema (3).

El presupuesto devengado para 2015 en medicamentos y dispositivos médicos en Ecuador fue de 221, 9 millones de $US, lo cual representa 9% del presupuesto total del Ministerio de Salud Pública (MSP) (4). Por este motivo, es imprescindible asignar dicho gasto a intervenciones que sean costo-efectivas y que impacten de manera positiva en la prevención o recuperación del estado de salud de la población, para lograr así la optimización de los recursos disponibles del sector salud.

Como respuesta a esta necesidad de información de calidad para la toma de decisiones, en los años sesenta nació la evaluación de tecnologías. Inicialmente, dicha evaluación se orientaba hacia temas tan variados como la perforación de petróleo en alta mar, pesticidas, contaminación por automóviles, centrales nucleares, aviones supersónicos y el corazón artificial (5).

En las décadas de los sesenta y setenta, ya se habían analizado tecnologías en salud mucho antes de la aparición de la evaluación de tecnologías sanitarias entendida como tal. A mediados de los años setenta, el National Research Council de los Estados Unidos de América realizó evaluaciones de tecnologías y publicó los primeros informes sobre fertilización in vitro, predeterminación del sexo en los niños, retraso del envejecimiento, modificación del comportamiento humano por neurocirugía, estímulos eléctricos o materiales farmacéuticos (5).

La evaluación de tecnologías sanitarias (ETS) es la valoración sistemática de las propiedades y efectos de las tecnologías de la salud, que aborda los efectos directos e intencionados de estas tecnologías, así como sus consecuencias indirectas y no intencionales, y se dirige principalmente a informar la toma de decisiones respecto a su incorporación y difusión en los sistemas de salud (6). Una dimensión importante en la ETS es la económica, que se dirige a conocer cómo los resultados de salud generan diferente consumo de recursos mediante la realización de varios tipos de evaluaciones: costo-minimización, costo-utilidad, costo-efectividad y costo-beneficio. (7). Estas evaluaciones proporcionan información relevante para la formulación de políticas sanitarias efectivas y seguras, optimizando los recursos disponibles de un sistema de salud.

La ETS ha avanzado sustancialmente en las últimas décadas en el mundo (8–9), aunque solo a partir de 2012 se inició en Ecuador (10). Según el Acuerdo Ministerial 1034 del 30 de marzo de ese mismo año, se publicó el Estatuto Orgánico de Gestión Organizacional por Procesos del MSP (11) y la responsabilidad de realizar ETS se asignó a la Dirección Nacional de Inteligencia de la Salud (DIS). En 2013, la Organización Panamericana de la Salud (OPS) aprobó una resolución que apoya la ETS y su papel en la cobertura universal en salud (12).

Estas evaluaciones se han convertido en una herramienta de gran utilidad para las autoridades del MSP de Ecuador en el proceso de toma de decisiones en salud y se consideran una estrategia técnica generada como consecuencia de la reforma del sector salud en el país. Como una de sus atribuciones, la DIS emite informes técnicos sobre medicamentos que se encuentran bajo un proceso de análisis destinado a autorizar su compra y que no constan en el Cuadro Nacional de Medicamentos Básicos (CNMB) (13). Estas solicitudes son enviadas por los comités de farmacoterapia de los establecimientos de salud a la Dirección Nacional de Medicamentos y Dispositivos Médicos (DNMDM) del MSP, que analiza el cumplimiento de los requisitos establecidos por ley para iniciar el proceso de análisis para la compra de medicamentos que no están incluidos en el CNMB (15).

Los expedientes son revisados por el equipo de la DNMDM y aquellas solicitudes que requieren un análisis adicional son sometidas a una evaluación por parte de la DIS. Dicha dirección desarrolla tres tipos de informes, que se describen a continuación.

Primero, los Criterios Técnicos Basados en Evidencia, que se fundamentan en preguntas que tengan definidas la población, la intervención, el comparador y las medidas de resultado. Con estos términos se articula una estrategia de búsqueda de información científica relevante sobre el tema en la bibliografía médica. La búsqueda en estos casos es más limitada que la que se lleva a cabo en una ETS. El tiempo de respuesta aproximado es de 15 días laborables.

Segundo, la ETS corta, que corresponde a un trabajo de búsqueda sistematizada de bibliografía científica que dará lugar a un informe en el cual se evalúa la eficacia y seguridad de una tecnología sanitaria. El método para realizar estos informes se encuentra descrito en el Manual Metodológico de Evaluación de Tecnología Sanitaria de la Dirección de Inteligencia de la Salud.

Tercero, las ETS completas, que se realizan mediante un método descrito en el Manual Metodológico de Evaluación de Tecnología Sanitaria de la DIS. En esta evaluación se consideran, además de la eficacia y seguridad de la tecnología, otros aspectos, como su eficiencia, que se estima por medio de una evaluación económica.

Sobre la base de la información y las recomendaciones que contienen estos informes, se toma una decisión tras un proceso de priorización con la DNMDM. Los informes realizados por la DIS pueden ser favorables o desfavorables respecto a la eficacia y seguridad relativas del medicamento evaluado, es decir en relación con la eficacia y seguridad de la mejor alternativa disponible en el CNMB.

Cuando en el análisis de la DIS se ha demostrado que el medicamento tiene valor terapéutico añadido (incremental, es decir, respecto la mejor alternativa disponible), se considera pertinente realizar una evaluación económica que lo acompañe y que consiste en un análisis de impacto presupuestario desde la perspectiva del subsistema público como financiador. A continuación, el informe pasa a la DNMDM como un insumo no vinculante para la toma de decisiones.

El objetivo de este artículo es conocer el uso de la ETS en la toma de decisiones del MSP de Ecuador para la compra de medicamentos que no se encuentran en el CNMB.

## MATERIALES Y MÉTODOS

Los dos actores que participan en el proceso de toma de decisiones para la compra de medicamentos fuera del CNMB son la DIS y la DNMDM. Como el MSP inició la ETS en 2012, la información recopilada para este artículo corresponde al período 2012-2015.

La DIS cuenta con una base de datos en la cual se registran todos los informes realizados a partir del 2012. Esta base permite categorizar los informes por tipo de tecnología sanitaria, tema, solicitante, fecha de solicitud, fecha de entrega y categoría. En el presente análisis se analizan todos los informes registrados entre enero de 2012 y diciembre de 2015 en la base de datos de la Gestión Interna de Evaluación de Tecnología Sanitaria de la DIS.

La DNMDM emite informes de aprobación o denegación de las solicitudes de adquisición de “medicamentos especiales” tras los pedidos realizados por las unidades de salud. Se consideran medicamentos especiales aquellos que “no constan en el Cuadro Nacional de Medicamentos Básicos (CNMB) vigente, y que fueren necesarios en la Unidades de Salud,” siempre y cuando se incluya, primero, una solicitud en la cual se exprese el requerimiento del o de los medicamentos con la respectiva justificación técnica emitida por su Comité de Farmacoterapia y, segundo, un formulario de evaluación para solicitar la autorización de adquisición (16).

**CUADRO 1.** Distributión por año de los informes de evaluación de tecnologías sanitarias solicitados por la Directión de Inteligencia de la Salud según su finalidad y su situatión

Año	Utilizados para la compra medicamentos especiales	Proceso de solicitud no completado por la entidad de salud	En evaluación	Utilizados para guías de práctica cínica
2012	0	0	0	0
2013	11	11	0	0
2014	15	2	0	6
2015	10	0	3	1
Total	36	13	3	7

***Fuente:*** Repositorio de evaluación de tecnologías sanitarias de la Dirección de Inteligencia de la Salud.

## RESULTADOS

Según el registro de la Gestión Interna de ETS de la DIS, se han realizado 227 informes entre enero del 2012 y diciembre del 2015. Los informes están clasificados según las categorías de tecnologías sanitarias propuestas por la International Network of Agencies for Health Technology Assessment (INAHTA) (17), que incluyen medicamentos, dispositivos médicos y equipos, procedimientos médicos, programas sanitarios, y otras tecnologías.

De los informes realizados por la DIS, 87 corresponden a solicitudes de evaluación de eficacia y seguridad de medicamentos, 36, a dispositivos médicos, 29, a procedimientos médicos, 34, a programas sanitarios, y 41, a otras tecnologías sanitarias.

Los 87 informes se dividen en aquellos solicitados por el Viceministerio de Gobernanza de la Salud o el Despacho Ministerial (28 informes) y los pedidos por la DNMDM (59 informes). Todos sirvieron para informar el proceso de toma de decisiones, si bien sólo los solicitados por la DNMDM constituyen un insumo para la compra de medicamentos fuera del CNMB.

De los informes realizados para la DNMDM, 36 (61%) se hicieron para informar decisiones sobre la compra de medicamentos especiales. Hay 7 informes de medicamentos realizados por la DIS que se utilizaron directamente para la incorporación de medicamentos en guías de práctica clínica. Se consideró en evaluación a los 3 informes que en el momento de la recolección de datos se encontraban en proceso de análisis. El motivo por el cual no se utilizaron 13 informes es que la entidad solicitante no cumplió con todos los requisitos contemplados en el Acuerdo Ministerial 3155 relativos al proceso de envío de la solicitud (14).

Los informes realizados por la DIS aportan resultados y recomendaciones que pueden ser favorables o desfavorables respecto a la valoración de la eficacia y la seguridad del medicamento analizado en comparación con la mejor alternativa disponible en el CNMB. Al evaluar todas las decisiones tomadas por la DNMDM y compararlas con los resultados de los informes de la DIS, 29 de las 36 (80%) decisiones tomadas par ambas entidades fueron congruentes entre sí.

En 2013 y 2014 se tomaron dos decisiones que no coinciden y que corresponden a un informe, que, además de incluir los resultados de un análisis de impacto presupuestario, se demostraba la eficacia y seguridad superiores de un medicamento. La decisión final fue no autorizar la compra del medicamento solicitado.

En 2015, hubo dos casos en los cuales se demostraba la eficacia y seguridad relativas superiores de un medicamento que la DNMDM decidió no comprar sustentando su decisión en los resultados del análisis de impacto presupuestario. Se debe tener en cuenta que en estos casos las decisiones corresponden al análisis realizado en la DNMDM y que en la actualidad para la toma de decisiones sobre incorporación de nuevos medicamentos en Ecuador no se ha fijado aún un umbral de coste-efectividad incremental (*incremental cost-efectiveness threshold*), como ocurre en otros países.

## DISCUSIÓN

Según el análisis realizado en este artículo, la concordancia entre las decisiones tomadas por la DNMDM y los informes de la DIS alcanza 80%. Esto demuestra que los informes solicitados sobre los nuevos medicamentos sí se consideran para la toma de decisiones finales sobre su incorporación en el CNMB.

El uso de la ETS constituye una herramienta que las Autoridades del MSP utilizan de hecho para tomar decisiones de compra e incorporación de nuevos medicamentos basadas en pruebas científicas. Por consiguiente, cuando se desarrolló el Acuerdo Ministerial para la compra de medicamentos fuera del CNMB, la ETS se incorporó como un elemento importante adicional para la toma de estas decisiones.

Las ETS realizadas con medicamentos son las más frecuentes. Esto se debe a que actualmente las solicitudes de ETS provienen en su mayoría de la DNMDM. Es importante que la información generada en estos informes no solo se utilice para medicamentos, sino para los restantes tipos de tecnologías sanitarias. En 2016, la DIS ha llevado a cabo un proceso de difusión a todas las unidades del MSP sobre la ETS para ampliar las solicitudes de informes de ETS a todas las tecnologías médicas.

El 61% de utilización de los informes se debe considerar como un dato que no refleja la realidad actual. Si se considera por años (ver la tabla 1), se puede apreciar que en 2015 no se enviaban solicitudes incompletas a la DIS, porque ya se disponía tanto de una revisión previa de las mismas por parte de la DNMDM, como de un análisis conjunto que ambas direcciones realizaban de cada solicitud antes de iniciar la redacción de sus informes. Si se excluyen los datos de solicitudes incompletas, la utilización de los informes para la toma de decisiones alcanza 100%, tanto para la compra fuera del CNMB, como para su utilización en guías de práctica clínica.

Es importante tener en cuenta que la mayoría de los esfuerzos realizados en ETS por el MSP se dirigen hacia la compra de medicamentos especiales. Se debe reevaluar este proceso, considerando que la DIS está dejando fuera acciones tan importantes como el apoyo para la revisión del CNMB, la monitorización de las tecnologías incorporadas y otras actividades relacionadas. Generar una monitorización del uso de la ETS para saber qué se debe mejorar o promocionar respecto a su utilización por las autoridades, no solo del MSP sino del Sistema Nacional de Salud Ecuatoriano, es indispensable para poder demostrar la importancia de la toma de decisiones basada en la evidencia.

Aunque lo ideal sería la realización de estudios de costo–efectividad, actualmente se sólo realizan análisis de impacto presupuestario, si bien las evaluaciones económicas se pretenden incorporar en la toma de decisiones en el transcurso de 2016.

Los resultados de este estudio indican que, si bien se han hecho avances destacables, queda un largo camino por recorrer. Todo el esfuerzo invertido a lo largo de estos años de experiencia en la utilización de la ETS tiene que ir a la par de una amplia difusión de su utilidad en la toma de decisiones en política sanitaria por parte de las autoridades, así como de una coordinación estrecha entre las distintas instancias del MSP para priorizar los temas y las tecnologías médicas que han de analizarse en función de las necesidades del MSP, toda vez que aún no se utilizan métodos de priorización explícita ni formal en evaluación.

La incorporación de la ETS en la toma de decisiones basadas en la evidencia por parte de las Autoridades tiene como meta la sostenibilidad y la eficiencia del Sistema Nacional de Salud. Garantizar la importancia del papel y la función de la ETS en la toma de decisiones en relación con todas las tecnologías sanitarias por la Autoridad Sanitaria es la mejor forma de conseguirlo.

Al escribir este artículo ya existen otras iniciativas de instituciones tanto públicas como privadas relativas a la redacción de informes de ETS y es patente el interés en colaborar con las Autoridades de manera que sus actividades sean complementarias en un campo de acción que aún ha de ser explotado.

Considerando que la mayoría de informes evalúan medicamentos, es indispensable que se desarrollen informes para otros tipos de tecnologías y que se difunda masivamente su desarrollo y uso. A pesar de que el nivel de concordancia entre las decisiones es elevado, es importante que se continúen fortaleciendo las capacidades y la calidad de los informes, así como monitorizar la incorporación y difusión de las tecnologías autorizadas y financiadas para conocer la efectividad y el impacto de la ETS en Ecuador.

### Declaración.

Las opiniones expresadas por los autores son de su exclusiva responsabilidad y no reflejan necesariamente los criterios ni la política de la *RPSP/PAJPH* o de la OPS.

## References

[1] 1. Infante A, de la Mata I, López-Acuña D. Reform of health systems in Latin America and the Caribbean: situation and trends. Rev Panam Salud Pública Pan Am J Public Health. 2000;8(1-2):13-20. 10.1590/s1020-49892000000700005 11026771

[2] 2. Organización Panamericana de la Salud. La evaluación de tecnologías en salud en América Latina y el Caribe: Colección de casos. Washington, DC: OPS; 1999. Disponible en: http://apps.who.int/medicine-docs/documents/s16583s/s16583s.pdf Acceso el 5 de enero de 2016.

[3] 3. Malo-Serrano M, Malo-Corral N. Reforma de salud en Ecuador: nunca más el derecho a la salud como un privilegio. Rev Peru Med Exp Salud Pública. 2014;31(4):754-61. 25597730

[4] 4. Ministerio de Finanzas del Ecuador. Ejecución del presupuesto del Ministerio de Salud Pública. Quito: Ministerio de Finanzas; 2015. Disponible en: http://www.finanzas.gob.ec/el-presupuesto-general-del-estado/ Acceso el 6 de enero de 2016.

[5] 5. Goodman C. Introduction to health technology assessment. Falls Church, VA: The Lewin Group; 2014. Disponible en: https://www.nlm.nih.gov/nichsr/hta101/HTA101FINAL7-23-14.pdf Acceso el 6 de enero de 2016.

[6] 6. INAHTA. Disponible en: www.inahta.org Acceso el 5 de enero 2016.

[7] 7. Puig-Junoy J, Ortún-Rubio V, Pinto-Prades JL. Los costes en la evaluación económica de tecnologías sanitarias. Aten Prim. 2001;27(3):186–9. 10.1016/S0212-6567(01)78795-1 PMC7675957 11262325

[8] 8. Menon D, Topfer LA. Health technology assessment in Canada. A decade in review. Int J Technol Assess Health Care. 2000;16(3):896–902. 10.1017/s0266462300102168 11028144

[9] 9. Hinnovic. Health technology assessment across the world. Quebec: Hinnovic; 2007. Disponible en: http://www.hinnovic.org/health-technology-assessment-across-the-world/ Acceso el 25 de abril de 2016.

[10] 10. Ministerio de Salud Pública del Ecuador. Evaluación de tecnologías sanitarias. Boletín ETES. No. 1. Disponible en: http://www.salud.gob.ec/wp-content/uploads/downloads/2014/08/Bolet%C3%ADn-ETES-Ecuador-N0011.pdf Acceso el 25 de abril de 2016.

[11] 11. Ministerio de Salud Pública del Ecuador. Estatuto por Procesos del Ministerio de Salud Pública. Quito: Ministerio de Salud Pública; 2012. Disponible en: http://instituciones.msp.gob.ec/dps/morona_santiago/images/stories/PDF/LOTAIP/2%20Informacion%20Legal/Normas%20de%20Regulaci%C3%B3n/ESTATUTO%20POR%20PROCESOS%20DE%20MINISTERIO%20DE%20SALLTD%20PUBLICA.pdf Acceso el 3 de marzo de 2016.

[12] 12. Organización Panamericana de la Salud. Evaluación e incorporación de tecnologías sanitarias en los sistemas de salud. 28^a^ Conferencia Sanitaria Panamericana. Washington, DC: OPS; 2012. Disponible en: https://www.google.com/url?sa=t&rct=j&q=&esrc=s&source=web&cd=1&ved=0ahUKEwjJwpuD_J70AhWI-1B4KHTvjBbsQFggeMAA&url=http%3A-%2F%2Fwww.paho.org%2Fhq%2Findex.php%3Foption%3Dcom_docman%26task%3Ddoc_download%26gid%3D18487%26Itemid%3D%26lang%3Des&usg=AFQjCNEYY8IKkAfc2hGcLq0XUvoOfg3yRg&sig2=xRxZIDcP5sJ6AZSnxEJog&cad=rja Acceso el 3 de marzo 2016.

[13] 13. Consejo Nacional de Salud, Comisión Nacional de Medicamentos e Insumos, Ministerio de Salud Púbica. Cuadro Nacional de Medicamentos Basácos y Registro Terapéutico. 9^a^ Revisión. Quito; Consejo Nacional de Salud, Comisión Nacional de Medicamentos e Insumos, Ministerio de Salud Púbica; 2014. Disponible en: http://www.conasa.gob.ec/phocadownload/crimb9na/Cuadro_Nacional_de_Medicamentos_Basicos_9na_Revision.pdf Acceso el 3 de marzo de 2016.

[14] 14. Ministerio de Salud Pública del Ecuador. Instructivo para autorizar la adquisición de medicamentos que no constan en el Cuadro Nacional de Medicamentos Básicos. Quito: Ministerio de Salud Pública; 2013. Disponible en: http://www.controlsanitario.gob.ec/wp-content/uploads/downloads/2014/09/A-3155-Instructivo-para-autorizar-la-adquisici%C3%B3n-demedicamentos-que-no-constan-en-el-CNMB-p.pdf Acceso el 26 de febrero 2016.

